# Electrically switchable polymer stabilised broadband infrared reflectors and their potential as smart windows for energy saving in buildings

**DOI:** 10.1038/srep11773

**Published:** 2015-07-01

**Authors:** Hitesh Khandelwal, Roel C. G. M. Loonen, Jan L. M. Hensen, Michael G. Debije, Albertus P. H. J. Schenning

**Affiliations:** 1 Functional Organic Materials and Devices, Department of Chemical Engineering and Chemistry, Eindhoven, University of Technology, Den Dolech 2, 5600 MB Eindhoven, the Netherlands; 2 Dutch Polymer Institute (DPI), P.O. Box 902, 5600 AX Eindhoven, The Netherlands; 3 Unit Building Physics and Services, Department of the Built Environment, Eindhoven University of Technology.; 4 Functional Organic Materials and Devices, Department of Chemical Engineering and Chemistry, Eindhoven, University of Technology, Den Dolech 2, 5600 MB Eindhoven, the Netherlands

## Abstract

Electrically switchable broadband infrared reflectors that are relatively transparent in the visible region have been fabricated using polymer stabilised cholesteric liquid crystals. The IR reflectors can change their reflection/transmission properties by applying a voltage in response to changes in environmental conditions. Simulations predict that a significant amount of energy can be saved on heating, cooling and lighting of buildings in places such as Madrid by using this switchable IR reflector. We have also fabricated a switchable IR reflector which can also generate electricity. These polymer based switchable IR reflectors are of high potential as windows of automobiles and buildings to control interior temperatures and save energy.

A number of studies report an increasingly strong relation between the indoor environmental quality of buildings and the health, productivity and well-being of building occupants^1^,^2^,^3^. In many cases, extensive use of heating, ventilation and air-conditioning (HVAC) equipment is needed to ensure that requirements for desired indoor conditions are met. This energy-intensive form of space conditioning, however, not only leads to high utility bills but also causes carbon dioxide emissions and other negative side-effects for the environment^4^.

Building envelope technologies play a major role in making the built environment more sustainable^5^. In particular, windows are important design elements, as they balance access to solar gains and thermal transmission with the need for a view to the outside and consequent energy consumption for heating, cooling and artificial lighting^6^,^7^. A number of recent innovations have opened up new opportunities for building envelopes as a flexible compromise between these competing design objectives. These include shutters^8^, blinds^9^, electro- and photo-chromic windows^10^,^11^,^12^,^13^,^14^,^15^,^16^, light responsive systems^17^, liquid crystal based windows^18^,^19^ and thin inorganic coatings^20^. A limitation to these technologies is that they either cannot alter their properties with changing environmental conditions (leading to an increase in energy demand) or they absorb/scatter light in the visible region, reducing the visual contact between occupants and the outside environment (resulting in extra energy for artificial lighting to maintain the interior illumination level^21^) or both.

Autonomous organic based temperature responsive infrared (IR) reflectors which reflect infrared light at elevated temperature and transmit it at lower temperature^22^,^23^ and a manually controlled electrically switchable infrared/visible reflector based on an inorganic material (tin doped indium oxide nanocrystals in niobium oxide glass) have been reported^24^. Simple narrowband cholesteric IR reflectors (with reflection bandwidths of 100–200 nm) have also been fabricated to help reduce the thermal transmission through a window^25^,^26^. However, these reflectors only can reflect a limited fraction of incident solar light, which reduces their impact. Furthermore, some of them interfere with visible light, requiring the use of artificial lighting to compensate.

Switchable broadband infrared reflecting/transmitting windows which can reflect a large amount of infrared solar energy in summer and transmit it in winter appear to be very attractive^27^,^28^,^29^. Such windows should be transparent over the entire visible light region (400–700 nm) so that extra energy is not required to maintain the illuminance level of the room^30^. Infrared light from the sun spans from 700 nm to ~1 mm; however, more than 75% of the energy of infrared light lies between 700 to 1400 nm^31^.

In this article we report fabrication of electrically switchable broadband infrared reflectors based on the properties of cholesteric liquid crystals (Ch-LC) and study its impact on energy saving in buildings. Such an integrated approach combining fabrication of switchable infrared reflector with simulation studies has not been reported before. The fabricated broadband switchable IR reflector can be switched manually between IR reflective and transmissive modes on applying an electric voltage to respond to changes in environmental conditions without interfering with the visible region. We have predicted the impact of this switchable IR reflecting window on energy savings in office buildings in various climates across the globe: using infrared reflectors in an office building in Madrid, Spain, environment could save more than 12% of the energy used on heating and cooling without any increase in energy consumption for lighting. We have also taken first steps towards the fabrication of a switchable IR reflector that can simultaneously act as an electricity generating window.

## Materials and Methods

### Materials

**Figure 1:**
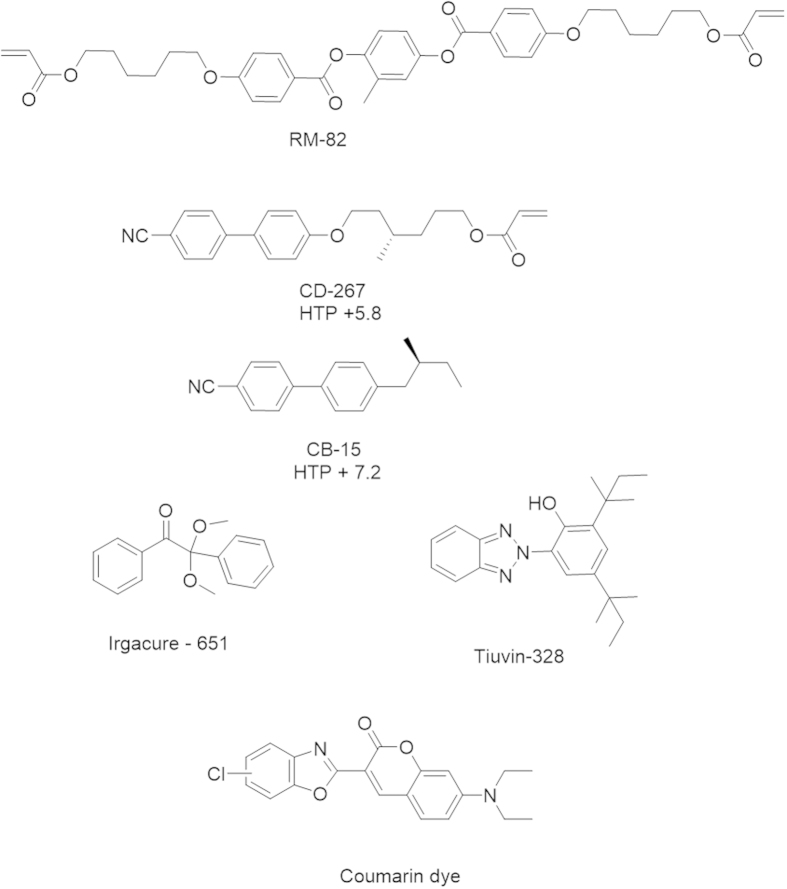
Molecular structures of the materials used.

Chiral dopant CD-267 was obtained from Philips Research Lab. Nematic liquid crystal mixture E7 (∆ε = +14.3) and chiral dopant CB-15 ((S)-4’-(2-Methylbutyl)[1,1’-biphenyl]-4-carbonitrile) were purchased from Merck. Photoabsorber Tinuvin-328 and photoinitiator Irgacure-651 were purchased from Ciba Specialty Chemicals Ltd (Fig. 1). Indium Tin Oxide (ITO) coated rubbed polyimide cells with 20.4 μm gap thickness were purchased from Instec Inc.

### Methods

Nematic liquid crystal E7 (70.2 wt %), chiral dopants CD-267 (13.8 wt %) and CB-15 (12.1 wt %), UV absorbing dye Tinuvin-328 (2.0 wt %), photoinitiator Irgacure-651 (1.2 wt %) and cross-linker RM-82 (0.7 wt %) were mixed in a glass vial by stirring continuously for one hour at room temperature. For the fabrication of electricity generating IR reflector, 0.25 wt % Coumarin based dye was added to the above mixture. These mixtures were used to fill cells via capillary action. These were then photopolymerized with UV light of intensity ~7 × 10^−5^ W/cm^2^ at room temperature for 60 min.

## Results and Discussion

### Fabrication of the broadband infrared reflector

**Figure 2:**
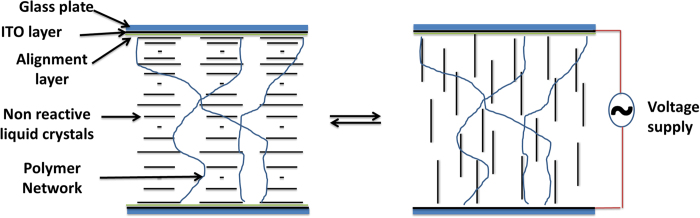
Schematic diagram shows the planar state (left) and homeotropic state on applying the electric voltage (right).

Ch-LCs are known to reflect light as a result of their self-organized molecular helices^32^,^33^,^34^. The so-called pitch of the helix determines the wavelength of reflection. The reflection bandwidth is normally limited to ~75 to 100 nm depending on the central wavelength and the birefringence^35^. A polymer stabilized Ch-LC approach was previously used for the fabrication of electrically switchable mirrors and optical components in the visible region^36^,^37^,^38^,^39^,^40^,^41^,^42^,^43^,^44^,^45^,^46^. In this research the polymer network is used to create a broadband IR reflector and to stabilize the planar orientation of the nonreactive LC component (Fig. 2). Monoacrylate (CD-267) and diacrylate (RM-82) molecules were chosen to fabricate a lightly cross-linked network (Fig. 1). The nonreactive liquid crystal mixture (E7) was chosen as it has high dielectric anisotropy (∆ε = +14.3) which allows the molecules to switch easily to the homeotropic state on applying an electric voltage. Upon removing the electric voltage, orientation of the molecules reverts back to the original state in presence of an alignment layer and polymer network.

**Figure 3:**
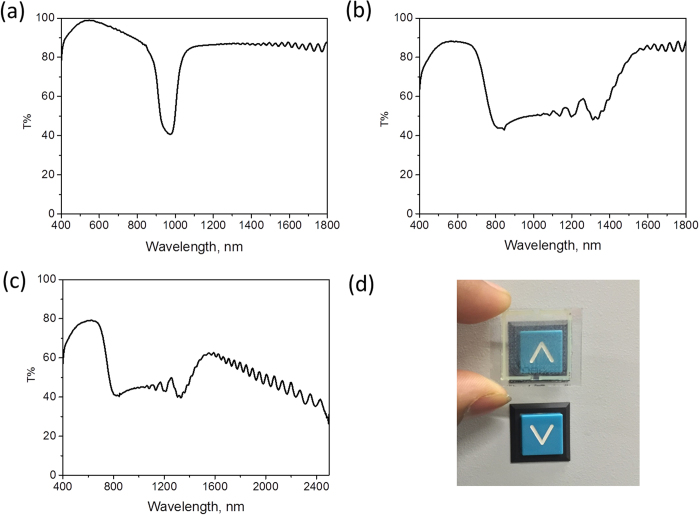
Transmission spectra of cholesteric liquid crystal gel **a**) before polymerization **b**) after photo-polymerization with the influence of the ITO coated glass plates omitted and **c**) after photo-polymerization including the absorbance of ITO and **d**) Photograph of the IR reflector demonstrate the transparency in the visible region.

To induce a cholesteric phase, chiral dopants CD-267 and CB-15 were added to the host nematic liquid crystal mixture. To fabricate the broadband IR reflector, a concentration of chiral dopant was chosen such that the reflection peak of the Ch-LC mixture was centered around 1000 nm. Figure 3a shows the transmission spectrum of the Ch-LC gel before polymerization. The reflection peak of the mixture was found to be centered around 970 nm with a typical bandwidth of 105 nm. To achieve the broadband IR reflection, a pitch gradient was attained using photoinduced diffusion during photopolymerization^19^,^47^. A UV-intensity gradient was generated by adding 2.0 wt % of the UV-absorber Tinuvin- 328 to the mixture. Upon UV irradiation, the intensity gradient induces the chiral monoacrylate (monofunctional) molecule to undergo faster polymerization at the top compared to the bottom of the sample. This depletion of monoacrylate at the top causes diffusion of monoacrylate molecules from the bottom towards the top of the film. Thus, diffusion of monoacrylate leads to non-uniform distribution of chiral dopant (monoacrylate) in the liquid crystal gel and thus a pitch gradient is generated^35^,^48^. Figure 3b shows the transmission spectrum corrected for the electrode layers (ITO), to emphasize changes in the active reflecting material of the liquid crystal gel after polymerization. The Ch-LC polymer gel reflects a broader band of infrared light from 700 to 1400 nm while remaining predominantly transparent in the visible region (88.5% transmission at 550 nm, Figs. 3b,d). The ITO layer on the glass plate absorbs in the infrared region (Supplementary Fig. S1) as can be seen in the non-normalized (with respect to the ITO plate) transmission spectrum (Fig. 3c). However, absorption of ITO does not have a significant impact on energy savings in a building because it only absorbs light primarily >1300 nm.

### Electrically switchable infrared reflector

**Figure 4:**
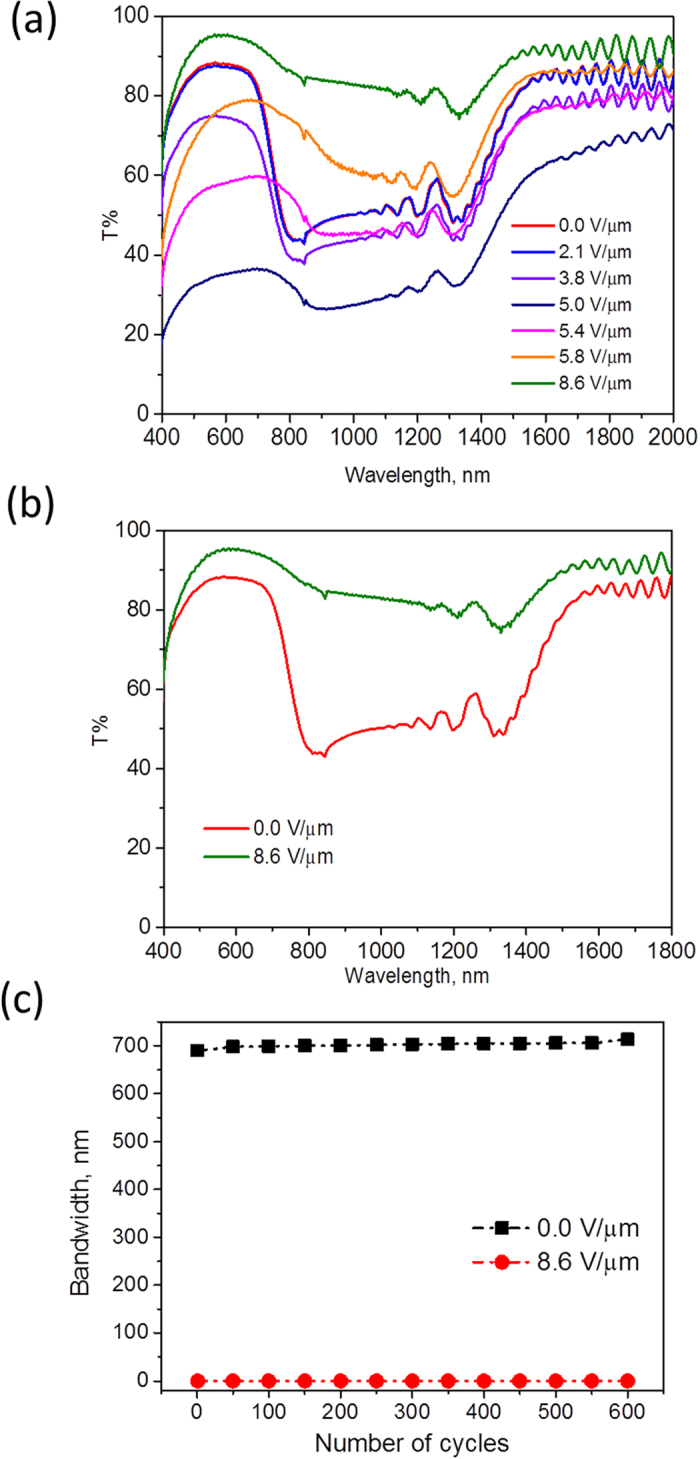
**a**) Transmission spectrum of IR reflector on varying the applied voltage from 0 V/μm to 8.6 V/μm **b**) Transmission spectrum of cholesteric gel in reflective and transmissive states at 0 V/μm and 8.6 V/μm, respectively **c**) Switching measurements shows stable bandwidth after 600 cycles between 0 V/μm and 8.6 V/μm.

After successfully fabricating the infrared reflecting gel, we have studied the switching behaviour by applying an electric voltage. At zero to approximately 2.1 V/μm, the gel exhibits a broad infrared reflection band with high transparency in the visible region. On further increasing the voltage (e.g. 5.4 V/μm), scattering in the visible region increases while reflection in the infrared remains approximately the same (Fig. 4a). This could be attributed to tilting of the cholesteric liquid crystal yielding most likely a focal conic state along with some unwinding of the helix, resulting in more scattering^49^,^50^. Such a situation (at 5.4 V/μm) could be useful as a ‘privacy’ state of a window device, effectively controlling IR transmission while simultaneously causing a scattering state which is translucent but not transparent. On further increasing the voltage to 8.6 V/μm, the liquid crystal gel becomes transparent to infrared as well as visible light (Fig. 4b). This effect is attributed to homeotropic orientation of the E7 molecules^50^,^51^,^52^.

To use the IR reflector for window applications, it is important for the device to be able to switch many times without altering the transmissive or reflective qualities. We have not observed any significant change in the transmission spectrum even after switching between 0 V/μm and 8.6 V/μm for 600 cycles (Fig. 4c, S2).

### Simulation study

On employing the cholesteric liquid crystal gel between the glass panes of windows, a significant fraction of infrared light can be reflected (0 V/μm) or transmitted (8.6 V/μm) depending on the applied voltage, without significantly affecting the transmission in the visible region (Fig. 4b). To understand the potential impact of this electrically switchable infrared reflector on energy demand in a building, we simulated a medium size office building (Supplementary Table S1), with properties as defined by the U.S. Department of Energy as the model using Trnsys simulation software (TRNSYS v 17- Transient system simulation tool, 2013). In these dynamic whole-building performance predictions, the effect of the switchable IR reflector on potential primary energy savings for heating, cooling and artificial lighting was analyzed^53^. In the simulation study we have not included the energy required to switch the gel from reflective to transmissive state (vide infra). The window was switched to the transparent state when the indoor operating temperature was lower than 22 °C during daytime. In this way, solar heat gains are allowed to the building when it is cold, but reflected when there is a risk of indoor overheating.

**Figure 5:**
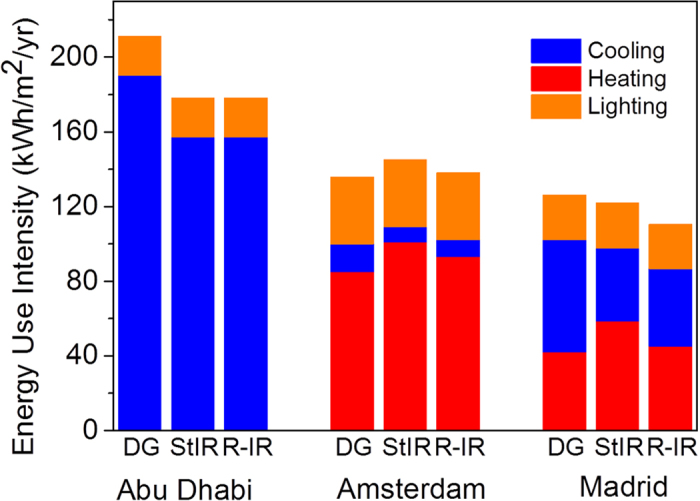
Comparison of energy use intensity for a normal double glazed window (DG), static IR reflector (StIR) and the switchable (responsive) infrared reflector (R-IR) for three different climates.

Three different climates were chosen to understand the relation between environmental conditions and energy savings in buildings by using a switchable IR reflector: (1) Abu Dhabi, United Arab Emirates (2) Amsterdam, the Netherlands and (3) Madrid, Spain. The results are compared to a reference configuration employing normal double glazing (DG) for a south facing office. We have also compared the results of the switchable IR reflector (R-IR) with the static permanent broadband IR reflector (StIR) in the ‘off’ state. This evaluation demonstrates the potential impact a switchable system may have in comparison to static IR reflectors. The window properties that were used in the simulations were obtained using the calculation methods in the software Optics-5, 2013 (Supplementary Table S2). Our simulations suggest that the impact of switchable infrared reflectors in office buildings depends on the local environmental conditions. In Abu Dhabi, which has warm and sunny climate throughout the year, the application of a switchable IR reflector (R-IR, 178.1 kWh/m^2^/yr) leads to cooling energy savings of >15% compared to a normal double glazing window (DG, 211.2 kWh/m^2^/yr). However, in such locations, the demand for cooling is high throughout the year, which makes non-visible solar gains unwanted at all times. Given the current window switching control strategy, the window would be in the transparent state for only 23 hours of the year. Hence, similar energy savings are achieved with static broadband IR reflection (Fig. 5, StIR versus R-IR). In contrast, in the Amsterdam environment the window would be switched from the reflective state to the transmissive state for a considerable amount of time (1684 hours). The simulation results reveal that in heating-dominated climates such as Amsterdam, there is no need for either switchable or static IR reflectors. The decrease in warming of building interiors by solar heat gains would lead to an increase in demand of energy required for heating, offsetting any energy savings for heat rejection in warmer seasons.

In climates such as Madrid which have more seasonal influences than in Amsterdam or Abu Dhabi, trade-offs between heating and cooling are better balanced. The ability of the windows to switch between a transparent and a reflecting state thus becomes more interesting, as well-controlled solar gains have benefits during both heating and cooling periods. The total energy that can be saved by replacing the normal double glazing window (DG, 126.1 kWh/m^2^/yr) with the fabricated switchable IR reflector (R-IR, 110.6 kWh/m^2^/yr) is 12.3%. For the same situation, a static IR reflector (121.9 kWh/m^2^/yr) would only result in 3% energy savings. The climate in Madrid is predominantly sunny, yet has a relatively large number of cold days, so continual reflection of NIR sunlight is not always a good strategy. The window would need to be switched to the transparent state for an estimated 870 hours. It is advised that switchable IR reflectors are used in this case to utilize passive solar gains in a dynamic way.

The range of dynamic optical properties (Supplementary Table S1) is obtained by modifying only the transmission of non-visible sunlight; the visible transmittance (T_vis_) remains high. Therefore, the amount of energy consumption for interior illumination (lighting) by using switchable IR reflector is similar to the reference transparent double glazing window in all the climates studied (Fig. 5). The energy consumption for lighting makes a significant contribution (10–26%) of total building energy use, with variations caused by differences in climatic conditions. Depending on the operation strategy for manual or automated opening and closing of light-blocking shading systems, the electricity consumption for lighting could go up by a factor of 2 to 3^54^,^55^. On considering this concomitant effect on lighting energy use and comparing it to the case where blinds tend to be closed and lights are on^56^, the energy saving performance of switchable IR reflectors in locations like Madrid goes above 20%.

The performance potential for switchable IR reflectors in this study is compared on the basis of primary energy savings. During actual building operation, the implications of IR reflectors can be wider, because they reduce energy bills as well as carbon dioxide emissions, and can result in air-conditioning systems with smaller capacities. In addition, the controlled access of solar gains leads to better indoor environmental quality, due to more efficient daylight utilization, more view to outside, and lower radiant temperature of window surfaces which increases thermal comfort in summer^57^. All of these features have a positive effect on the health and well-being of the building inhabitants. However, a detailed analysis of all the costs and benefits, including the costs for switching, still needs to be carried out on the basis of a full-size window.

### Towards electricity generating switchable IR reflectors

The energy consumption on switching a window employing an IR reflector was determined by


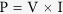


yielding a consumption of 3.74 mW (in the 10 mm × 10 mm cell) or 37.45 W/m^2^ (V is 8.6 V/μm and I is 21.4 μA) through the cell on applying voltage. To offset the electrical energy consumption of the switching, we have fabricated a switchable IR reflector which could also be employed as an energy generating window, collecting sunlight via embedded luminescent molecules and directing the luminophore emission to edge-mounted solar cells which could be used to eventually switch the window between IR reflective and transmissive states^58^,^59^. We have added 0.25 wt % of a fluorescent coumarin-based dye as a model system, which partially absorb light only in the visible light region, to the same cholesteric liquid crystal mixture. Photopolymerization of the dye incorporated Ch-LC was carried out as described earlier, resulting to reasonably similar reflection bands to the previous samples which did not include fluorescent dye (Fig. 3b).

**Figure 6:**
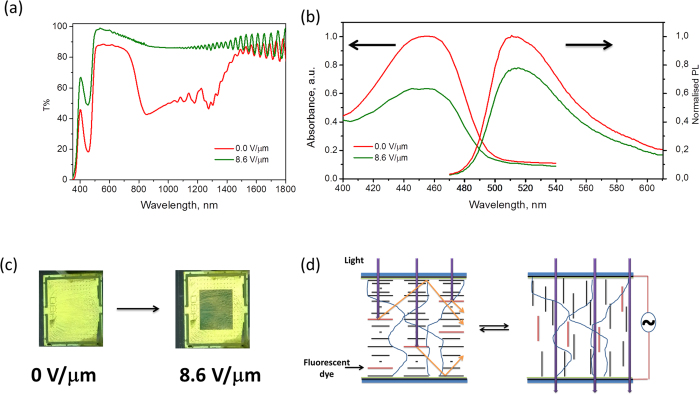
**a**) Transmission spectrum of the IR reflector containing 0.25 wt % coumarin-based dye in the mixture at 0 V/μm and 8.6 V/μm. **b**) Normalized absorption of the dye and edge emission from cell at 0 V/μm and 8.6 V/μm **c**) photograph of the Ch-LC gel with the middle of the cell in the planar (left) and homeotropic (right) state viewed through crossed polarizers. **d**) Schematic (ideal case) representing the orientation of the Ch-LC gel in the planar (left) and homeotropic (right) states and the absorption and waveguiding of sunlight.

Figure 6a shows the transmission spectrum of the IR reflector with the dye incorporated in the mixture at 0 V/μm (red spectrum). The absorption band of the coumarin dye is centered at λ = 455 nm. On applying an electric voltage (8.6 V/μm), the cholesteric polymer gel becomes transparent in the infrared region (Fig. 6a) and the peak absorption of the dye decreases by 37% (Fig. 6b). The decrease in the absorption suggests that the dye aligns with E7, yielding a homeotropic alignment on applying voltage. This was confirmed by observing the cell under crossed polarizers as it turns black on applying an electric voltage (Figs. 6c,d). When this glass plate was exposed to sunlight, an edge emission of the cell was observed at λ = 510 nm. The embedded dye absorbs sunlight and emits at longer wavelengths. A fraction of this emitted light is trapped in the glass plate which then guides the light towards the edges via total internal reflection^59^. Upon application of an electric voltage a decrease in edge emission was observed as less sunlight is absorbed. The edge emission decreases by 21% on changing the applied voltage from 0 V/μm to 8.6 V/μm (Fig. 6b). These experiments demonstrate that the window device is capable of absorbing and re-emitting sunlight from the visible range so that it reaches the edge of the lightguide in both the planar and homeotropic states. In principle, one could convert the edge emitted light into electrical current by attaching a photovoltaic cell to fabricate an electricity generating switchable IR reflector^18^.

## Conclusions

Electrically switchable broadband IR reflector has been fabricated by combining several simple procedures. The fabricated reflector can tune the reflection and transmission fractions of infrared light depending on the applied voltage while remaining predominantly transparent in the visible region. Simulation studies reveal that switchable IR reflectors are attractive in sunny, temperate climates with a relatively large heating demand in buildings, such as Madrid. It is predicted that more than 12% of energy can be saved using a switchable IR reflector compared to a normal double glazing window and 9.3% compared to a static IR reflector. The application of such IR reflectors is not limited to the windows of buildings; they also have other potential application such as in automobiles as it is transparent in the visible region. Finally, we describe the first steps to offset the electrical energy consumption in the switching. By incorporation of a fluorescent dye into the switchable IR reflector mixture, a luminescent solar concentrator can be fabricated in which the edge emission of the dye can be converted into electricity by a photovoltaic cell. In principle, such a window can be made transparent in the visible region^60^ and the generated electricity can be used for assisting switching from the IR reflective to the transparent state. Overall this integrated study, combining experimental results with simulations, shows the great potential of switchable broadband IR reflectors for energy saving.

## Additional Information

**How to cite this article**: Khandelwal, H. *et al*. Electrically switchable polymer stabilised broadband infrared reflectors and their potential as smart windows for energy saving in buildings. *Sci. Rep*. **5**, 11773; doi: 10.1038/srep11773 (2015).

## Supplementary Material

Supplementary Information
