# Supplementary material for: Electrically switchable polymer stabilised broadband infrared reflectors and their potential as smart windows for energy saving in buildings
Source: Sci Rep. 2015 Jul 1;5:11773. doi: 10.1038/srep11773 (PMC4487232; doi:10.1038/srep11773)
Supplement: Supplementary Information [file srep11773-s1.pdf]

Supplementary Information

Electrically switchable polymer stabilised broadband infrared reflectors and their potential as smart windows for energy saving in buildings

Hitesh Khandelwal, Roel C. G. M. Loonen, Jan L. M. Hensen, Michael G. Debije

and Albertus P. H. J. Schenning

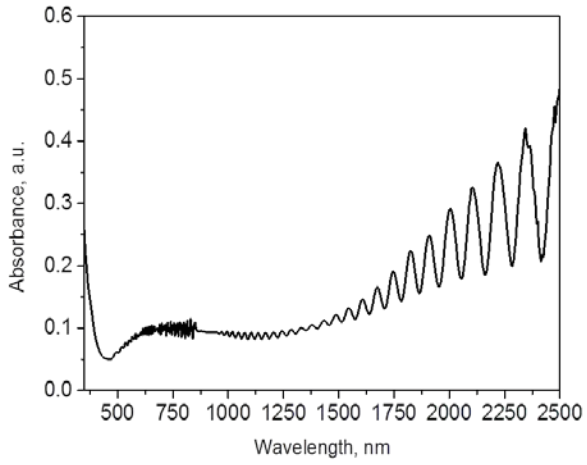

Fig. S1 Absorption spectrum of empty ITO coated cell.

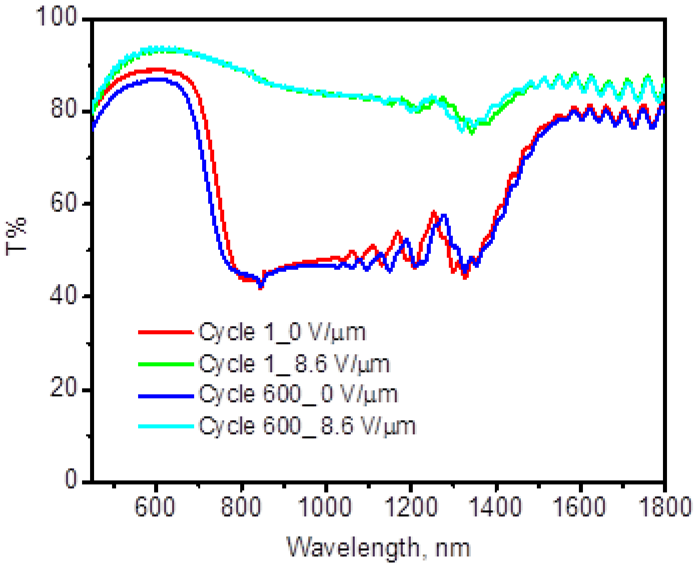

Fig. S2 Transmission spectrum of IR reflector before and after switching for 600 cycles.

Table S1 Details and assumptions of the case study building. See [53] for a full description.

**Building description**	
Window-to-wall ratio	48%
U-value window	1.3 W/m^2K
Opaque facade elements	Heavyweight, insulation according to ASHRAE 90.1-2010
Room depth	6.4 m
**Conversion to primary energy**	
Heating system efficiency	0.9
COP heat pump	3
Primary energy conversion factor	2.5
**Building usage scenario**	
Climate data	TMY2 weather files
Occupied hours	09:00 – 17:00 on weekdays
Occupant heat loads	5.6 W/m^2
Equipment loads	13 W/m^2
Lighting power density	9 W/m^2
Heating and cooling set point	20°C and 26°C

Table S2 Window properties of the double glazing units in the simulations.

**Type of Window**	T_{sol}	T_{vis}	**SHGC**
Reference	0.60	0.76	0.70
Switchable IR reflector – transparent state	0.59	0.72	0.68
Switchable IR reflector – reflecting state	0.48	0.69	0.56

T_{vis}: Transmission in the visible region
T_{sol}: Transmission in the whole solar region
SHGC: Solar heat gain coefficient
